# Artificial Intelligence in Nuclear Cardiac Imaging: Novel Advances, Emerging Techniques, and Recent Clinical Trials

**DOI:** 10.3390/jcm14062095

**Published:** 2025-03-19

**Authors:** Ilana S. Golub, Abhinav Thummala, Tyler Morad, Jasmeet Dhaliwal, Francisco Elisarraras, Ronald P. Karlsberg, Geoffrey W. Cho

**Affiliations:** 1 David Geffen School of Medicine, University of California, Los Angeles, CA 90095, USA; igolub@mednet.ucla.edu (I.S.G.); athummala@mednet.ucla.edu (A.T.); tmorad@mednet.ucla.edu (T.M.); jsdhaliwal@mednet.ucla.edu (J.D.); felisarraras@mednet.ucla.edu (F.E.); 2 David Geffen School of Medicine, Department of Cardiology, University of California, Los Angeles, CA 90095, USA; karlsberg@cvmg.com; 3 Cardiovascular Research Foundation of Southern California, Beverly Hills, CA 90048, USA

**Keywords:** cardiovascular disease (CVD), artificial intelligence (AI), nuclear medicine (NM), myocardial perfusion imaging (MPI), computed tomography (CT), single photon emission computed tomography (SPECT), positron emission tomography (PET), magnetic resonance imaging (MRI), coronary flow reserve (CFR), convolutional neural networks (CNNs), recurrent neural networks (RNNs), support vector machines (SVMs)

## Abstract

Cardiovascular disease (CVD) is a leading cause of death, accounting for over 30% of annual global fatalities. Ischemic heart disease, in turn, is a frontrunner of worldwide CVD mortality. With the burden of coronary disease rapidly growing, understanding the nuances of cardiac imaging and risk prognostication becomes paramount. Myocardial perfusion imaging (MPI) is a frequently utilized and well established testing modality due to its significant clinical impact in disease diagnosis and risk assessment. Recently, nuclear cardiology has witnessed major advancements, driven by innovations in novel imaging technologies and improved understanding of cardiovascular pathophysiology. Applications of artificial intelligence (AI) to MPI have enhanced diagnostic accuracy, risk stratification, and therapeutic decision-making in patients with coronary artery disease (CAD). AI techniques such as machine learning (ML) and deep learning (DL) neural networks offer new interpretations of immense data fields, acquired through cardiovascular imaging modalities such as nuclear medicine (NM). Recently, AI algorithms have been employed to enhance image reconstruction, reduce noise, and assist in the interpretation of complex datasets. The rise of AI in nuclear medicine (AI-NM) has proven itself groundbreaking in the efficiency of image acquisition, post-processing time, diagnostic ability, consistency, and even in risk-stratification and outcome prognostication. To that end, this narrative review will explore these latest advances in AI in nuclear medicine and its rapid transformation of the cardiac diagnostics landscape. This paper will examine the evolution of AI-NM, review novel AI techniques and applications in nuclear cardiac imaging, summarize recent AI-NM clinical trials, and explore the technical and clinical challenges in its implementation of artificial intelligence.

## 1. Introduction

Cardiovascular disease (CVD) is a leading cause of death worldwide and accounts for over 30% of annual global fatalities [1]. Ischemic heart disease, in turn, is a frontrunner of global CVD mortality (age-standardized rate of 108.8 deaths per 100,000 individuals). Prevalent cases of total CVD nearly doubled from 271 million in 1990 to 523 million in 2019, and the number of CVD deaths has steadily increased from 12.4 million in 1990 to 18.6 million in 2019 and more recently to 19.8 million in 2022 [1,2]. In the setting of these rapidly growing cases and coronary disease burden, understanding the nuances of cardiac imaging and risk prognostication becomes paramount.

**Figure 1:**
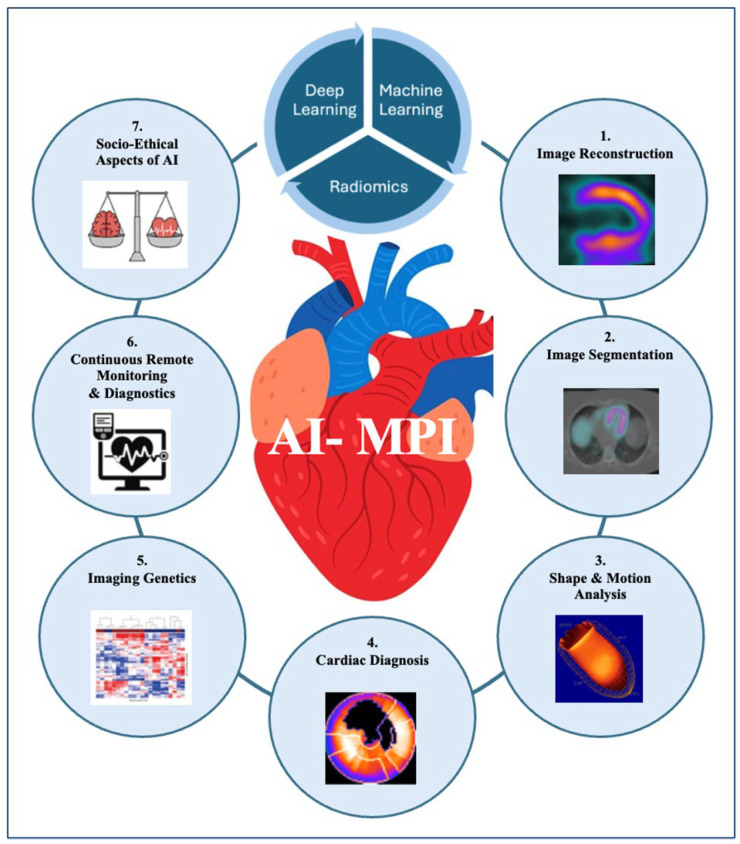
Artificial intelligence in nuclear cardiac imaging. This figure highlights key applications of artificial intelligence (AI) within myocardial perfusion imaging (MPI). AI in nuclear medicine facilitates enhanced imaging reconstruction, complex dataset interpretation, diagnostic accuracy, and risk stratification, as well as therapeutic decision-making in patients with cardiovascular disease.

Myocardial perfusion imaging (MPI) is a frequently utilized and well established testing modality due to its significant clinical impact in disease diagnosis and risk assessment [3,4,5]. Recently, nuclear cardiology has witnessed major advancements, driven by innovations in novel imaging technologies and improved understanding of cardiovascular pathophysiology [3,4,6,7]. Applications of artificial intelligence (AI) to MPI—by single photon emission computed tomography (SPECT) or positron emission tomography (PET)—have enhanced diagnostic accuracy, risk stratification, and therapeutic decision-making in patients with coronary artery disease (CAD) [3,4,6,8,9]. AI techniques such as machine learning (ML) and deep learning (DL) neural networks offer new interpretations of immense data fields, acquired through cardiovascular imaging modalities such as nuclear medicine (NM). Recently, AI algorithms have been employed to enhance image reconstruction, reduce noise, and assist in the interpretation of complex datasets. The REVEAL AI software, for example, has shown promise in automating the detection of ischemia and predicting patient outcomes with high accuracy, as evidenced by its performance in the REVEAL-CHF trial. Moreover, projects such as HeartEnsembleNet have explored the innovative hybrid ensemble approach for cardiovascular risk prediction, where AI is used for prognostic modeling in cardiac imaging [10]. Similarly, Federated Learning in Smart Healthcare offers a comprehensive review on the privacy, security, and predictive analytics with respect to IoT (a network of data exchanging objects or so-called “internet of things”) integration. In essence, this paper deals with machine learning techniques which facilitate our understanding of how AI models may be deployed in healthcare settings [11]. In summary, the rise of AI in nuclear medicine (AI-NM) has proven itself groundbreaking in the efficiency of image acquisition, post-processing time, diagnostic ability, consistency, and even in risk-stratification and outcome prognostication [3,4,5,6,8,9,12] (Figure 1).

This backdrop lays a critical foundation for this narrative review. Here, we will explore these latest advances in AI in nuclear medicine and its rapid transformation of the cardiac diagnostics landscape. To that end, this comprehensive review will first examine the evolution of AI-NM. Next, we will review novel AI techniques, including deep learning models, machine learning algorithms, and hybrid approaches. This review will cover applications of AI in nuclear cardiac imaging and discuss recent AI-NM clinical trials. Lastly, this manuscript will also explore the technical and clinical challenges in its implementation of artificial intelligence.

In summary, this review seeks to conciliate clinical reservations and synthesize growing amounts of research, to help encourage a comprehensive understanding of AI’s role within nuclear cardiac imaging. Here, we will highlight recent advances in nuclear cardiology and provide an overview of AI technology and clinical trials that have contributed to these innovations.

## 2. Evolution of Nuclear Cardiac Imaging: A Transition from Traditional to AI-Integrated

To better understand AI’s transformation of the cardiodiagnostics landscape, this review will first explore the evolution from traditional to AI-integrated nuclear medicine. The application of AI methods (i.e., deep learning and traditional machine learning models) into molecular imaging modalities such as PET and SPECT is an advancement that required years in the making. This transition from traditional methods to AI-integrated techniques involves a history of MPI advancements such as hybrid imaging modalities, novel radiotracers, and molecular techniques.

### 2.1. Hybrid Imaging Modalities

Fundamentally, nuclear medicine analyzes the uptake of radiopharmaceuticals to visualize, diagnose, and treat cardiovascular disease. With its focus upon the heart’s functional pathophysiology, NM empowers clinical detection of cardiac abnormalities at the molecular level [13]. NM modalities, such as positron emission tomography and single-photon emission computed tomography, employ the capture of gamma rays using a 2D planar gamma camera [14,15]. Radiopharmaceuticals emit gamma photons via direct gamma decay or indirect positron emissions, each of which triggers annihilation interactions that can be detected by their respective NM imaging system [15]. While the addition of SPECT to PET improves accuracy and sensitivity, utilizing both SPECT and computed tomography (CT) in tandem creates an anatomic reference and further enhances accuracy [14]. Recent strides in the traditional sphere of cardiac diagnostics enable precisely this: the integration of hybrid imaging modalities, such as PET/CT and PET/MRI (magnetic resonance imaging) [13,16]. These hybrid techniques, in turn, offer more comprehensive anatomical/functional cardiac assessments than what their counterparts can provide alone [16]. The PACIFIC trial (PET Assessment of Coronary Flow Reserve and Ischemic Burden for Improving Cardiovascular Outcomes) exemplifies this hybrid approach, demonstrating that PET-derived coronary flow reserve (CFR) can predict adverse cardiac events better than conventional MPI alone [17].

### 2.2. Novel Radiotracers

Alongside AI-integrated techniques, numerous advancements in nuclear cardiovascular imaging have also found their roots in the development and utilization of new radiotracers. Compounds such as 18F-flurpiridaz offer improved myocardial perfusion imaging, with higher resolution and reduced radiation exposure compared to traditional single photon emission computed tomography testing. 18F-flurpiridaz has a high first-pass extraction fraction (0.9), meaning its degree of tissue uptake is heavily dependent upon blood flow [18,19]. 18F-flurpiridaz’s long half-life (110 min) and excellent extraction fraction allow for tracer injection during exercise, outside the PET scanner room [20]. Thus, image acquisition can be delayed for some time. In contrast, stress PET studies which utilize traditional agents (i.e., O-15 H2O, N-13 NH3, and Rb-82) require radiopharmaceutical injection within the PET scanner itself. As a result, exercise stress has been nearly impossible to test, and myocardial perfusion PET has traditionally relied upon pharmacologic stress instead. The phase III study of 18F-flurpiridaz demonstrates superior image quality and diagnostic performance, potentially setting a new standard for MPI [18,19].

### 2.3. Molecular Techniques

Molecular techniques targeting specific biological processes (i.e., inflammation, fibrosis, and cardiotoxicity) have also created new avenues for early detection and personalized treatment of cardiac diseases. While current imaging modalities may assess the extent and pattern of myocardial scarring, they are often unable to detect fibrosis during a window when disease-modification remains possible. Now, groundbreaking NM methods such as gallium-68 fibroblast activation protein inhibitor positron emission tomography (68Ga-FAPI PET) offer imaging of fibrosis activity directly [21]. The TARGET trial (Targeted Imaging of Cardiac Fibrosis) highlights the potential of 68Ga-Galmydar in visualizing myocardial fibrosis, offering insights into disease progression and treatment response [21]. These advances empower early fibrosis detection, differentiation between active and inactive disease, and individualized patient care.

Other molecular imaging biomarkers aid in the personalized surveillance and detection of cardiotoxicities. Novel modalities offer higher sensitivity to detect subtle changes in cardiac homeostasis, as well as improved reproducibility and observer independence. Molecular techniques that detect early signs of cardiotoxicity include the following: 123I-metaiodobenzylguanidine SPECT which visualizes sympathetic innervation, 18F-FDG and somatostatin receptor (68Ga-DOTATOC/DOTATATE) PET which flag metabolic changes and inflammation, as well as 68Ga-fibroblast activation protein inhibitor PET which serves as a biomarker for cardiac remodeling [22].

These traditional methods—hybrid imaging modalities, novel radiotracers, and molecular techniques—are but a few advancements in the history and evolution of nuclear medicine. These strategies are themselves an innovation and steppingstone to radiomics and AI-integrated techniques. While radiomics offer the extraction and analysis of vast quantitative data from medical images, AI algorithms empower complex data processing to aid in image interpretation, treatment planning, response assessment, and, ultimately, the individualization of patient care.

## 3. Novel AI Techniques in Nuclear Cardiac Imaging

Next, we transition to discuss the role of artificial intelligence within nuclear cardiology. MPI using SPECT or PET is a commonly ordered nuclear imaging test in both the inpatient and outpatient setting. It aids in diagnosing coronary artery disease and evaluating the progression and presence of ischemia, as well as risk stratification. SPECT MPI is more commonly utilized than PET MPI as it is more widely available and cost-effective. However, PET is often more desirable as it may offer higher sensitivity when compared to SPECT for diagnosing coronary artery disease [23]. AI, in turn, has the potential to revolutionize several steps of the MPI workflow for both modalities, namely acquiring, reconstructing, and enhancing images; segmenting anatomical features; quantifying myocardial perfusion; predicting the presence of disease; risk stratifying; and relaying clinical findings. AI offers a significant time processing reduction with similar, or even superior, image interpretations [24] (Figure 1). A further discussion of AI model architectures and parameters, in the context of advanced computational science or AI engineering, however, is beyond the scope of this paper. Here, we will discuss two categories of AI involvement in nuclear medicine: deep learning (via convolutional neural networks and recurrent neural networks) and machine learning (via support vector machines and random forests).

### 3.1. Deep Learning Models

While PET is often viewed as superior to SPECT, both diagnostic techniques are confounded by high noise levels and low spatial resolution. These, in turn, necessitate significant reconstruction and enhancement processing following image capture [25]. Several AI deep learning models have already been utilized to enhance SPECT and PET images, including convolutional neural networks (CNNs) and recurrent neural networks (RNNs). CNNs and RNNs, respectively, possess their own advantages and limitations, and can be dovetailed to further enhance diagnostic capability.

#### 3.1.1. Convolutional Neural Networks

**Figure 2:**
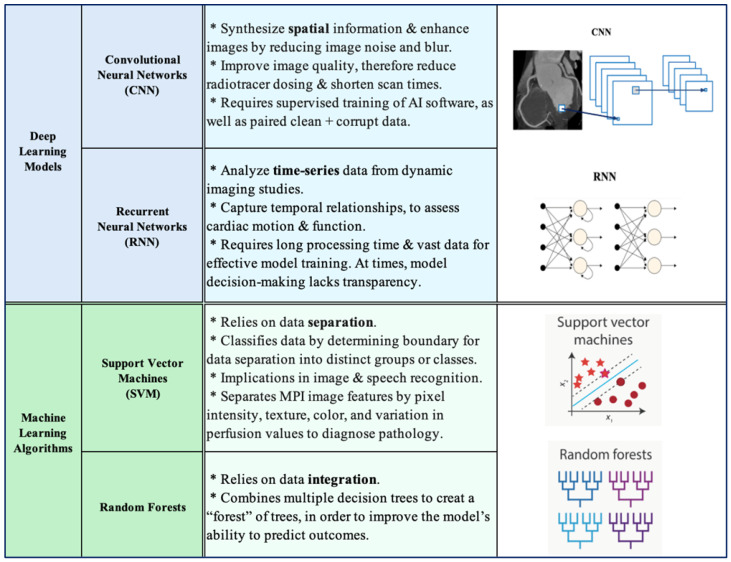
Deep learning models and machine learning algorithms.

Convolutional neural networks (CNNs) are a pillar in AI-enhanced cardiac imaging. CNNs assist in synthesizing spatial information and enhancing images by reducing image noise and blur (Figure 2). By significantly improving image quality, CNNs empower a reduction in radiotracer dosing and shorten scan times without compromising diagnostic accuracy [25]. One drawback of CNNs in clinical practice, however, is their need for supervised AI software training as well as paired clean and corrupt referencing data. This has inspired research into alternative models, both supervised and unsupervised, which may allow for fewer training hours.

#### 3.1.2. Recurrent Neural Networks

In contrast, recurrent neural networks (RNNs) are utilized for time-series data (Figure 2). Within the sphere of cardiac nuclear medicine, RNNs analyze time-series data from dynamic imaging studies including gated SPECT or PET. (Gated nuclear medicine studies are similar to their non-gated counterparts but possess the added capability to incorporate an electrocardiogram and assess myocardial perfusion, ejection fraction, and wall motion simultaneously). Because RNNs are highly capable in capturing temporal relationships, their streamlined incorporation into gated studies enables higher diagnostic yield. However, limitations do exist, including the following: computational complexity requiring long processing times, the need for extensive data to effectively train the model, model overestimation and overfitting, lack of transparency regarding model decision-making, and the need for trained clinicians capable of effectively utilizing the novel software (Figure 2).

While each deep learning model has its own limitations, RNNs can be integrated with CNNs to form hybrid models. This hybrid approach uniquely offers the optimization of both spatial and temporal data, to further enhance diagnostic accuracy while limiting individual model drawbacks. To date, few studies have utilized both learning models to enhance MPI; further investigation is needed to assess the efficacy and limitations of such hybrid approaches.

Several studies show the utility of deep learning algorithms in cardiac imaging and their translation to patient care. Interestingly, AI appears to be equally capable of reading cardiac imaging when compared to seasoned clinicians. Garcia et al., for instance, created an AI-driven report to compare the diagnostic accuracy between AI modeling and nuclear cardiology experts in patients who had undergone SPECT MPI. Garcia et al. found no significant difference in the diagnosis of coronary artery disease or ischemia [26]. This model uniquely tracks all steps in report generation and offers clinicians a lens into the algorithm’s decision making.

AI may be superior to its human counterparts in certain aspects, especially in terms of optimizing cardiographic diagnostic accuracy alongside reduced work hours. Kusumoto et al., for instance, developed a deep learning-based CNN model to diagnose coronary artery disease using SPECT MPI. Kusumoto’s team found that the AI-based system had the highest diagnostic performance, while also improving provider time spent [27]. Another single-center study by Feher et al. developed an algorithm which integrated both deep learning and machine learning models, to analyze the risk of heart failure admission in patients who underwent SPECT MPI. Their team uncovered a significant improvement in predicting heart failure admissions, when compared to a model using only clinical parameters [28]. Although this study was retrospective, utilizing AI to interpret MPI in real time might foster up-to-date diuretic augmentation and thus prevent hospitalization and reduce morbidity.

Deep learning AI models are also helpful with respect to PET analysis. Bors et al., for instance, created several CNN models to evaluate the risk of a major adverse cardiac event (MACE) in individuals who underwent [82-Rb] PET-based MPI. Their team demonstrated that AI-based models perform equivalently when compared to traditional quantitative MPI measurements (including stress Myocardial Blood Flow and Myocardial Flow Reserve) for MACE prognostication [29]. Because SPECT MPI is a more common form of cardiac nuclear imaging, more studies utilize AI-based models to enhance SPECT than PET. Additional research is needed to better characterize AI’s utility in PET MPI, as AI integration becomes more commonplace in cardiac imaging. These studies are paramount for the future of cardiac imaging and its relation to patient care, as clinicians are faced with rising clinical volume and demands.

### 3.2. Machine Learning Algorithms

Another significant breakthrough in AI has come in the form of machine learning (ML) algorithms. While deep learning models and machine learning algorithms are both under the umbrella of artificial intelligence, they have key differences. Deep learning can learn from its own mistakes without human intervention, whereas machine learning requires a human’s involvement to fix issues. The drawback to deep learning, however, is the significant computational power required to run effectively. Though neither type of AI algorithm requires supervised learning, unsupervised learning is more often prone to diagnostic errors especially when encountering new data. Similar to deep learning, several types of machine learning have shown promise in nuclear cardiac imaging, namely support vector machines (SVMs) and random forests.

#### 3.2.1. Support Vector Machine Learning

A SVM is a unique type of supervised machine learning algorithm that classifies data by finding the best possible boundary to separate data into distinct groups or classes (Figure 2). The SVM has powerful implications in both medical and non-medical realms, including image and speech recognition. In the world of nuclear cardiac image recognition, SVMs are being utilized to assist in identifying coronary artery disease and ischemia on SPECT and PET MPI. This AI technology has the impressive ability to separate MPI image features including pixel intensity, texture, color, and variation in perfusion values to more accurately diagnose pathology. Berkaya et al., for instance, developed an SVM machine learning algorithm to identify perfusion abnormalities on SPECT MPI and found that the model provided similar diagnostic accuracy to expert analysis [30]. SVMs have also shown superiority to other machine learning models in assessing coronary artery disease. Wang et al. referred patients with suspected coronary artery disease to [13]N-NH3 PET/CT myocardial perfusion imaging (MPI) and [18F-FDG] PET/CT myocardial metabolic imaging (MMI) and found that an SVM outperformed six other machine learning models in CAD detection [31].

#### 3.2.2. Random Forest Machine Learning

On the other hand, a random forest model is a type of machine learning algorithm that combines multiple decision trees, thereby creating a “forest” of trees to improve the model’s outcome prediction ability (Figure 2). While both SVMs and random forest models are examples of machine learning used to classify data, the models are inherently built differently: SVMs separate data while random forests integrate data. Given this contrast, random forests are often thought to be superior in their diagnostic capabilities. Erdagli et al. investigated several CNNs and machine learning models (including random forests and SVMs) and compared their respective performances in diagnosing coronary artery disease on nuclear MPI (PET and SPECT) and non-nuclear MPI (cardiovascular magnetic resonance imaging (CMR)) [32]. The study interestingly found that, of all the AI tools assessed (including CNNs and machine learning models), random forest remained superior in enhancing SPECT MPI in the diagnosis of coronary artery disease. The SVM was deemed second best, followed by the remaining machine learning and CNN models. This study also found that CMR imaging allowed for better diagnostic results than nuclear MPI imaging [32]. These key findings underscore the diagnostic capabilities of various imaging modalities and AI models. Most importantly, these findings delineate the importance of weighing each screening test and AI tool alongside their strengths and weaknesses, particularly when applied to patient care.

## 4. Applications and Current AI Technologies in Nuclear Cardiac Imaging

Thus far, we have explored the two primary categories of AI involvement in nuclear medicine: deep learning (via convolutional neural networks and recurrent neural networks) and machine learning (via support vector machines and random forests). Next, we transition from AI techniques to its applications. We explore applications in image reconstruction and enhancement, automated interpretation and diagnosis, quantitative analysis, and prognostic models.

### 4.1. Image Reconstruction and Enhancement

**Figure 3:**
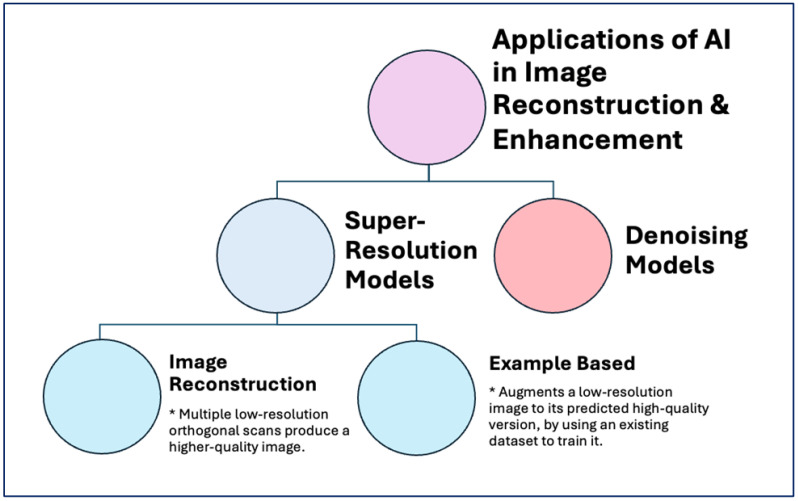
Applications of artificial intelligence in image reconstruction and enhancement.

Images may become corrupted with noise during acquisition, processing, or transmission. This issue is particularly significant in cardiac nuclear imaging, where factors such as inadequate breath depth, baseline arrhythmias, or motion during image acquisition are common causes of poor image quality and reduced utility. Image denoising, a classic topic in computer vision and a crucial part of image processing, aims to remove the “noise” captured during these stages (Figure 3).

However, denoising was time-consuming before the introduction of artificial intelligence and, in particular, deep learning models. Several deep learning-based methods have been proposed to overcome noise artifacts in cardiovascular imaging. One study by Sohlberg et al. [33] compared various denoising models using MPS (myocardial perfusion SPECT) images. They evaluated convolutional neural networks (CNNs), residual neural networks (RESs), UNET, and conditional generative adversarial networks (cGANs). These models were trained using ordered subset expectation maximization (OSEM) reconstructed MPS studies with different acquisition times. Researchers then compared the performance of these models against each other and against images that did not utilize deep learning-based denoising. Sohlberg et al. used coefficients of variation (CoV) and the structural similarity index measure (SSIM) to assess noise levels. Their findings demonstrated that all deep learning models outperformed OSEM, with cGAN demonstrating the most significant reduction in CoV in the myocardium [33].

In conjunction with denoising, super-resolution has also become increasingly prominent (Figure 3). Super-resolution often falls into two main categories: (1) image reconstruction through multiple low-resolution orthogonal scans, which produces a higher-quality image, and (2) example-based SR, which augments a low-resolution image to its predicted high-quality version by using an existing dataset to train it. Recent CMR, SPECT, and MPI advancements have enabled deep learning for denoising and super-resolution processing.

Xia et al., for instance, introduced a conditional generative adversarial network for 3D isotropic cardiac MR reconstructions using single-stack images: thereby eliminating the need for high-resolution or multiple low-resolution scans [34]. Xia et al.’s method (which employed a generator and discriminator with a hybrid loss function) achieved a Dice similarity coefficient of 0.95 for the left ventricle cavity and 0.81 for the myocardium, when comparing real and synthesized images. This approach enhances cardiac quantification, nonrigid registration, and ventricle segmentation. Ultimately, this modality provides accurate results without requiring longer acquisition times [34].

Zhao et al., in contrast, proposed a denoising super-resolution GAN (DnSRGAN) designed to reconstruct cardiac magnetic resonance (CMR) images [35]. Their approach combined a feed-forward denoising convolutional neural network (DnCNN) with a super-resolution generative adversarial network (SRGAN). They employed a loss function to monitor the GAN’s gradient descent, ensuring stable and efficient training while enhancing the quality of the generated images. The study demonstrated that the DnCNN effectively pre-denoised the CMR images, and the SRGAN further optimized the reconstruction using this improved input [35].

### 4.2. Automated Interpretation and Diagnosis

**Figure 4:**
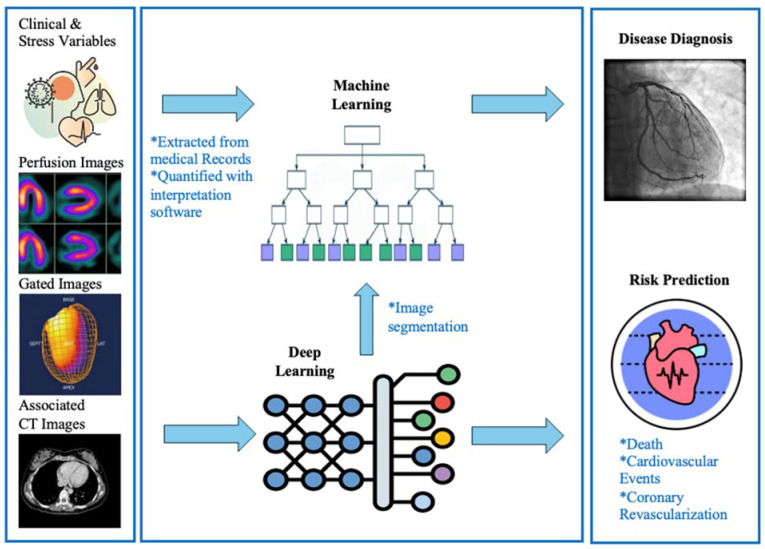
AI in establishing disease diagnosis and risk prediction. This figure demonstrates a stepwise approach, by which AI (artificial intelligence) manipulates clinical data towards patient-specific disease diagnosis and risk prediction. AI applications within nuclear medicine pool data from clinical and stress variables, perfusion images, gated images, and associated computed tomography (CT) images. Via AI modalities such as machine learning and deep learning, these applications offer important advances in disease diagnosis and risk adjudication.

Machine learning and deep learning AI technologies have made significant advancements in diagnosing various cardiac conditions (Figure 4). In particular, AI holds significant potential in facilitating coronary artery disease detection. AI offers an impressive ability to rapidly integrate data from nuclear medicine with other imaging modalities used in chronic coronary syndrome detection, such as echocardiography, cardiac magnetic resonance, and CCTA [36].

AI technologies are particularly adept at detecting subtle deviations which clinicians might miss, thus enhancing diagnostic accuracy. Interestingly, they perform similarly to expert clinicians with regard to specific diagnoses. In one study, the effectiveness of multi-input machine learning was compared to the diagnostic capabilities of medical experts in identifying CAD [37]. The researchers used invasive coronary angiography as the gold standard while implementing InceptionV3 and Random Forest algorithms. They analyzed MPI combined with clinical data, achieving an accuracy of 78.43% for the models, close to the 79.15% accuracy achieved by medical experts. The agreement between the automated models and the experts was about 86%, suggesting that integrating image processing and clinical data can produce results comparable to those of human diagnosticians [37].

Others have focused on using deep learning techniques to analyze T1 mapping in cardiac magnetic resonance (CMR) imaging and measure myocardial interstitial fibrosis. This approach is vital to identify nuanced patterns of fibrosis and their specific disease correlate: cardiomyopathies, ischemia, and arrhythmias [38]. Antonopoulos et al. validated one such machine learning methodology to differentiate between healthy and diseased myocardium based on stable radiomic features [39]. This study emphasized the importance of analyzing shape, intensity, and texture within radiomic data to enhance diagnostic performance for disorders like hypertrophic cardiomyopathy (HCM) and amyloidosis [39].

The integration of innovative techniques is also underway. Wang et al. introduced an innovative cine-based screening and diagnostic method for cardiovascular disease (CVD) utilizing a video-based Swin Transformer (VST) instead of traditional convolutional neural networks (CNNs) [40]. This method included two models: one for initial screening and another for diagnostics. The screening model achieved an area under the curve (AUC) of 0.986 and an F1 score of 0.977, outperforming individual cine views. The diagnostic model also performed excellently, with an AUC greater than 0.96 for various CVD classes. Testing on external datasets confirmed the reliability of this method. Significantly, the VST model surpassed traditional CNNs and matched the performance of board-certified physicians while processing results substantially faster [40]. It particularly excelled in diagnosing pulmonary arterial hypertension (PAH) in CMR-negative patients better than the experts, showcasing AI’s capability in identifying subtleties.

### 4.3. Quantitative Analysis

Like AI’s application to image reconstruction and automated interpretation, its role in facilitating quantitative analysis is a key field of interest. The measurement of perfusion and structural function, in particular, has become a significant focus within cardiovascular imaging using ML/AI. Traditionally, these modalities rely on manual operator input, which can be cumbersome and introduce variation. This limitation, in turn, is an area for which ML/AI holds major potential.

A study conducted by Sun et al. [41] evaluated deep learning methods for fully automating the segmentation of the left ventricle (LV) from 4D flow MRI. The study involved 101 subjects, including 75 patients who had experienced a myocardial infarction. Researchers acquired thin slices from a short-axis cine stack and obtained 4D flow MRI using echo-planar imaging during standard clinical protocols. They created RAW and SAX masks from the 4D flow MRI and applied deep learning models to assess the impact of data preprocessing and network structures on segmentation performance. The CNN model SAX2DF demonstrated excellent LV segmentation, achieving an average Dice coefficient of 84.5% and an average surface distance of 3.14 mm compared to the ground truth [41]. This model also yielded the lowest uncertainty score and the highest correlation for left ventricular ejection fraction (LVEF), end-diastolic volume (EDV), and end-systolic volume (ESV), suggesting its potential reliability in clinical settings [41].

One group of researchers presented a video-based DL algorithm (EchoNet-Dynamic) that surpassed human expert performance in segmenting LV, assessing cardiomyopathy, and estimating EF. This algorithm uses expert human tracing for supervised learning. Interestingly, expert evaluators often preferred the model’s predictions to human annotators [42]. The model’s automatic LV segmentation may prove helpful for interjection into workflow and incorporation into clinical practice. Other studies have focused on demonstrating the ability of DL models to automatically annotate 2D videos, with reproducible accuracy to manual measurements by expert sonographers [43]. These studies are a revolutionary step towards the automation of cardiac function evaluation.

### 4.4. Prognostic Models

Recent advancements in ML and AI have driven significant developments within the healthcare sector itself, particularly in clinical practice. A multicenter study involving coronary computed tomography angiography (CCTA) across 11 sites showcased this innovation by employing a deep learning convolutional neural network (CNN) to segment coronary plaque [44]. The model (known as “ConvLSTM”) demonstrated an impressive 87% agreement with expert CAD-RADS classifications, indicative of strong consistency [44]. This automated plaque quantification process might significantly reduce interpretation variability, making it an invaluable tool for physicians in the near future.

In another noteworthy study by Miller et al., ML techniques were utilized to predict abnormal myocardial perfusion among 20,418 patients from SPECT MPI and an additional 9019 from an external cohort [45]. By leveraging extreme gradient boosting (XGBoost), the ML model outperformed traditional methods like the Diamond–Forrester approach [45]. This highlights the potential of ML to enhance prediction models, thereby aiding clinicians in imaging and treatment decisions.

Additionally, prediction models have been used to extract features that may indicate mortality risk [46]. One group focused on patients with newly diagnosed pulmonary arterial hypertension (PAH). It employed a multilinear principal component analysis-based ML approach to identify mortality and survival features from cardiac magnetic resonance (CMR) short-axis images. Their findings improved the 1-year mortality prediction of the REVEAL score by pinpointing high-risk features throughout the cardiac cycle at the population level [46].

Furthermore, CMR ML analysis has been applied to predict arrhythmic events in patients with post-infarction scar burden and major adverse cardiac events (MACE). In a retrospective study of patients in the DERIVATE registry, researchers found that their ML models could quantify myocardial scars without user input, enhancing the prediction of MACE [47]. Notably, this ML quantification of dense and total scar offered improvements over conventional human assessments, facilitating better risk stratification for identifying suitable candidates for implantable cardioverter-defibrillators (ICDs).

## 5. AI Nuclear Medicine Clinical Trials

Various clinical trials have studied the applications and efficacy of AI-NM, most prominently in quantifying coronary artery disease and estimating MACE. Many of these trials have been discussed in previous sections, and a summary of the most notable may be found in Table 1.

As Table 1 describes, The Registry of Fast Myocardial Perfusion Imaging with Next-Generation SPECT (REFINE-SPECT) registry is a multicenter, international registry of longitudinal clinical and imaging data from patients who underwent MPI with SPECT [48]. As of 2024, this registry includes 45,252 patients and has allowed for the development and testing of various AI models.

In one study utilizing this registry, Hu et al. developed an ML model that included clinical, imaging, and stress test variables from patients with suspected CAD who underwent left heart catheterization (LHC) within 6 months [49]. This model better predicted early coronary revascularization when compared to total perfusion deficit (TPD) at the per-vessel and per-patient level and cardiologist interpretation at the per-patient level. In another study, Betancur et al. developed a DL model trained with raw and quantitative polar maps from patients without known CAD who underwent stress MPI [50]. Their model outperformed TPD at both the per-vessel and per-patient level, when assessed via area under the receiver-operating characteristic curve (AUC). More recently, Otaki et al. developed a DL model that included stress myocardial perfusion and wall motion, wall thickening maps, left ventricular systolic and diastolic volumes, age, and sex from 3578 patients in this population with suspected CAD [51]. Their model outperformed standard perfusion metrics and cardiologist interpretation (AUC 0.83 vs. 0.78 and 0.71), further supporting AI’s application in estimating CAD from MPI.

The REFINE-SPECT registry has also been used to build models that estimate MACE. Singh et al. included 20,401 patients who underwent SPECT-MPI to create a DL model that both predicted risk of all-cause mortality or non-fatal myocardial infarction and provided the clinician with an attention map that highlighted the vascular regions associated with increased risk [52]. The DL model included polar map image inputs of raw perfusion and gated derived maps of motion, thickening, phase angle, and amplitude in addition to age, sex, and end-systolic and diastolic volumes. While the model was validated on the REFINE population, it was tested on an external population of 9019 patients to establish external validity. The DL model significantly outperformed TPD metrics and a logistic regression model in estimating MACE over a 4.4-year study period. In this study population, patients with a score in the highest quartile had a 10-fold increased risk of MACE when compared to those with a score in the lowest quartile. Various other studies have been conducted in this subject and are summarized in Table 1 [53,54,55].

In 2013, Arsanjani et al. created a ML algorithm that included total perfusion deficit metrics, age, sex, and post-electrocardiogram prediction of CAD from a population of 1181 MPI studies [56]. Arsanjani’s algorithm demonstrated the potential to improve diagnostic efficacy when compared against both standard TPD and expert cardiologist interpretation. In 2018, Betancur et al. similarly demonstrated superior efficacy of a combined ML model in estimating 3-year MACE, when compared to physician review and automated perfusion measurements [37].

While the previously listed studies directly compared AI models against TPD or cardiologist interpretation, other models (as listed in Table 1) demonstrated high predictive value in identifying abnormal MPI as determined by expert review. This emerging body of evidence demonstrates a growing role for both ML and DL models in clinical application and interpretation of MPI.

## 6. Challenges and Limitations

While the advent of new advances in AI-NM offers the opportunity for clinical breakthroughs, these developments are not without their limitations. On a fundamental level, supervised AI software must be trained to perform a task: a process which is inevitably biased per the beliefs of the individuals and protocols involved. Unsupervised AI learning (including ML and DL algorithms) may not face this particular limitation, but they have the potential to introduce diagnostic and technical errors. This can be illustrated through denoising, whereby machine learning algorithms predict normal-dose imaging from acquired lower-dose images. One virtual imaging trial showed that while DL-based denoising appeared to perform superiorly through a fidelity-based figures of merit lens, ROC analysis showed that the algorithm introduced false perfusion defects and eliminated true defects from a detection-task perspective [57]. This represents a significant barrier in implementing deep learning scripts to real patients: where false positives and negatives hold significant repercussions on clinical testing and end-outcomes.

Other limitations in AI technologies arise from issues with generalizability and implementation. Several published AI-NM studies were performed using simulated patient populations or data from singular nuclear medicine machines. Even when accounting for simulated population demographic variations, this strategy creates inherent limitations for generalizability to real patient populations and studies performed on a multitude of machines. Simulated patients also lack the clinical complexity and nuance of real patients, who may have an extensive CAD history, are status-post multiple stent placements, or have undergone bypass surgery. AI studies which draw from multicenter databases often exclude these patients from analysis, though they represent a group of patients who might benefit most from these developments.

Moreover, as algorithms become more advanced, associated costs, particularly at the onset of new programs, will inevitably also increase. It is important to note that many of this review’s cited studies were conducted at large academic centers, each with advanced technology and expertise. An inherent challenge of AI-NM is implementing these artificial solutions in community hospital settings or those with more limited resources. AI in nuclear cardiology represents cutting-edge technology that is typically reserved only for large academic centers with sufficient capital to fund these projects. Limitations surround not only cost and accessibility but also the availability of practitioners trained in advanced AI-NM techniques in community settings. Practical implementation would require immense planning with regard to integration into existing clinical workflows, staff training requirements, and cost benefit analyses. Overcoming these barriers necessitates a stepwise approach, with incremental implementation in the years to come. Ultimately, much work remains to be conducted to apply machine learning algorithms to medically complex patients and improve the accessibility–affordability of AI-NM in a diverse clinical sphere.

We must also consider the ethical ramifications of AI’s rapid growth into the nuclear cardiology sphere. Like the development of AI within any field of medicine, questions arise regarding liability and implications of machine learning in replacing the expertise of trained imaging specialists. Despite goals for efficiency and cost-effective care, the ultimate priority for clinicians remains patient safety and well-being. Much additional research is needed in improving AI algorithmic function and transparency, mitigating biases in supervised learning, and optimizing extensive data training. All of this must be further explored before the general patient population acquires confidence in delegating their health to machine learning.

## 7. Future Directions

Despite its limitations, the AI-NM field is rapidly growing. It holds potential for several developments in the near future, ranging from technological advancements to new AI workflows which enhance the patient experience (Figure 4). As discussed previously, denoising and super-resolution are already being applied to reduce image artifacts and improve image quality. Next steps might include expanding these quality checks from simulated patient populations to real-life, diverse patient panels. Other strides might involve updating generative networks to include physical constraints, which could reduce the effect of slice misalignment and motion artifacts when analyzing MR cine images [58].

Image reconstruction is another domain where AI has been shown to assist clinical decision making but previously required vendor software to reconstruct attenuation-corrected images. New approaches using DL CNNs have been utilized via a post-reconstruction attenuation correction (PRAC) approach to create AC images independent of vendor software or raw projection data [59]. Many research projects that explore these topics are currently underway. For example, Abdulrazzaq et al. have recently investigated the consequential advancements of self-supervised learning in deep learning contexts and neural network training [60]. This paper offers a broader context on DL advancements, which is foundational to understanding and applying AI modalities.

As detailed above, the accessibility of AI technologies poses a significant limitation to their implementation, but streamlining data analysis by eliminating third parties and extra processing steps could expand the utility of these cutting-edge resources to a wider array of health systems. Another area of interest for additional research involves efficiency and patient experience. Researchers and clinicians alike are continually searching for opportunities to refine imaging protocols and reduce logistical burdens that both providers and patients face. As neural networks become more advanced, acquisition time and radiation doses can be further reduced to optimize the patient experience, while any quantification errors can be recovered with deep learning techniques [34]. This same approach can be adopted to AI algorithms which optimize scanner time and radiation exposure. For example, future work might combine DL-constructed AC images with stress-only imaging protocols, to optimize radiation exposure with diagnostic efficiency [61].

To date, much of the work in the AI-NM sphere has been performed with SPECT. As new data highlight the increased sensitivity of PET, however, the application of deep learning methods to PET myocardial perfusion imaging will inevitably also increase. ML algorithms have already been applied to cardiac PET and CT-based calcium scoring, and studies demonstrate that ML approaches are comparable to expert readers in assessing the degree of obstructive CAD [62]. Other research has excitingly explored the utility of machine learning in obtaining CAC scores from CT-attenuated correction scans performed for ischemic evaluation alone. Simply, this approach eliminates the need for an additional dedicated calcium scoring CT scan [63]. These automatically derived CAC scores are independently associated with MACE and highlight a direct clinical utility of novel AI applications [64]. Future work focused on fine-tuning these ML algorithms holds significant promise in reducing imaging burden for patients and easing the large quantity of reads for radiologists and cardiac imaging specialists.

Finally, an exciting direction for AI-NM is the sphere of personalized medicine. ML algorithms can process patient-specific clinical and imaging parameters, to output an individualized cardiac risk assessment. As discussed earlier in this review, Slomka et al. began working on this endeavor with the REFINE SPECT registry. This team amalgamated data from thousands of patients from numerous clinical sites to develop a database for use in diagnosis and prognostic predictions [34]. Similar work is currently underway; integrating PET MPI imaging data with patient demographic and clinical data in ML models can predict individual risk for MACE [64]. These AI algorithms allow for detailed and high-throughput analysis of patient data and MPI polar maps, at a level and speed beyond even the most experienced human reviewer. Perhaps, eventually, a patient might undergo a single nuclear cardiology study and—with the help of machine learning algorithms—instantaneously receive a personalized assessment of their cardiovascular health and risk for CVD.

**Table 1:** Clinical trials in AI-NM from 2013 to 2023.

Author, Year	N	Study Aims	Model Type	Model Input	Model Results
Arsanjani et al., 2013 [55]	1181 MPI studies	Prediction of obstructive CAD > 70% as assessed by coronary angiography	ML	TPDs, age, sex, and post-EKG CAD probability	Diagnostic accuracy (87.3% ± 2.1%) equal or better than expert readers; AUC (0.94 ± 0.01) greater than both TPD alone and expert readers (*p* < 0.001)
Betancur et al., 2018 [50]	1638 patients (9 centers)	Prediction of obstructive CAD as assessed by coronary angiography	DL	Raw and quantitative polar maps	AUC greater than TPD both at level of per-person and per-vessel (0.80 vs. 0.78 and 0.76 vs. 0.73, respectively; *p* < 0.01)
Betancur et al., 2018 [50]	2619 patients	Prediction of MACE	ML	28 clinical, 17 stress test, and 25 imaging variables	MACE prediction higher than expert diagnosis, stress TPD, and ischemic TPD (0.81 vs. 0.65, 0.73, and 0.71, respectively, *p* < 0.01)
Betancur et al., 2019 [56]	1160 patients (4 centers)	Prediction of obstructive CAD as assessed by coronary angiography	DL	Polar maps	AUC higher than TPD at per-patient (0.81 vs. 0.78) and per-vessel (0.77 vs. 0.73, *p* < 0.001) levels
Hu et al., 2020 [59]	1980 patients	Prediction of early coronary revascularization	ML	18 clinical variables (e.g., BMI), 9 stress test variables (e.g., peak heart rate), and 28 imaging variables (e.g., rest ejection fraction)	At per-vessel level, AUC (0.79, CI 0.77 to 0.80) greater than all TPD metrics (*p* < 0.001). At per-patient level, AUC (0.81, CI 0.79–0.83) greater than all TPD metrics (*p* < 0.001)
Apostolopoulos et al., 2021 [37]	566 patients	Prediction of flow-limiting CAD as assessed via angiography	DL	Clinical variables and polar maps	Accuracy rivaled expert diagnosis (78.4% vs. 79.2%)
Liu et al., 2021 [54]	37,243 patients (1 center)	Prediction of normal vs. abnormal MPI as assessed by expert review	DL	MPI-derived circumferential count profile maps and 6 clinical variables	AUC higher than TPD (0.87 vs. 0.84, *p* < 0.01) with higher accuracy (82.7% vs. 78.5%) and specificity (84.9% vs. 77.5%), though lower sensitivity (74.4% vs. 79.8%)
Otaki et al., 2022 [51]	3578 patients (9 centers)	Prediction of obstructive CAD as assessed by coronary angiography	DL	Stress myocardial perfusion, wall motion, and wall thickening maps with left ventricular volumes, age, and sex	AUC greater than stress TPD or expert reader (0.83 vs. 0.79 or 0.71, *p* < 0.0001) within dataset. Within external population, AUC also greater than stress TPD or expert reader (0.80 vs. 0.73 or 0.65, *p* < 0.001)
Singh et al., 2023 [52]	29,420 patients total (internal and external groups)	Prediction of death or non-fatal MI	DL	Myocardial perfusion, motion, thickening, and phase polar maps with age, sex, and cardiac volumes	AUC greater than logistic regression model or ischemic TPD in external population (0.70 vs. 0.65 or 0.63, *p* < 0.01). Calibration via Brier score 0.79 in internal group and 0.70 in external group

## 8. Conclusions

Myocardial perfusion imaging is an increasingly utilized platform, with a growing clinical impact upon cardiac disease diagnosis and risk prediction. Novel applications of AI to SPECT and PET mark a huge achievement in nuclear cardiology advancements. AI presents the opportunity for enhanced diagnostic accuracy, risk stratification, and therapeutic decision-making in patients with coronary artery disease. The rise of AI in nuclear medicine has proven itself groundbreaking in the efficiency of image acquisition and reconstruction, post-processing time, and in the interpretation of immense data fields. To that end, this review has highlighted these latest advances in AI in nuclear medicine and their rapid transformation of the cardiac diagnostics landscape. We have covered the evolution of AI-NM, novel AI techniques and applications in nuclear cardiac imaging, recent AI-NM clinical trials, as well as the technical and clinical challenges in implementing artificial intelligence.

Cardiac PET and SPECT are imaging modalities which capture a high bandwidth of data, and AI-assisted data processing demonstrates great promise in comprehensive image analysis. Understanding the nuances and challenges of AI-NM promotes a model for higher quality and individualized decision-making, prognostication, risk analysis, and cardiovascular outcome prediction. This review has sought to address clinical uncertainties and synthesize growing amounts of research to help encourage a homogenous approach to AI-assisted nuclear medicine. Utilization of AI does not intend to replace physician contribution but rather enhance it via imaging improvements which drive preventative, periprocedural, and interventional stages of cardiovascular care. AI-NM offers a key opportunity to enhance cardiovascular care in an increasingly advanced and data-driven sphere of modern medicine.
